# Commercialism

**Published:** 1916-06

**Authors:** J. P. Buckley


### COMMERCIALISM.

BY J. P. BUCKLEY, PH.D., D.D.S.

Any profession or business, in its upward or downward progress, naturally drifts along certain definite lines; and those who have watched closely the progress of dentistry must admit that in the past—not necessarily the recent past, but in the past, there has been and is a tendency toward commercialism. Has this tendency been detrimental to the progress of dentistry, or is it more in evidence today than it has been in the past? These are questions which it is but right and proper to consider. Therefore, let us discuss frankly, here in the open, this problem of commercialism; for a problem it certainly is, and one, which when settled, must be settled along broad and equitable lines. Some one has said, ''No question is ever settled until it is settled right.'' There is just a little manifest tendency on the part of some ultra-conservative members of our profession to criticise other members for what is termed ''commercial tendencies.'' Unless explained this term means nothing, but it is one that is frequently used. If we read the history of dentistry as recorded by our historians today, we learn of many men who spent their time, money, energy, and their lives working on some problem, the solution of which was of inestimable value to their profession, and, in many instances, to humanity. Many of these men were so absorbed in their work that they neglected their practice; and when they reached the sunset of life and were incapacitated for work, they found themselves dependent upon the charity of friends. We need not go beyond the membership of our own society to find striking examples of such lives. With these examples before them, other men who have worked equally as hard and in some instances have been able to accomplish more, have felt it a duty incumbent upon them to provide, as they went along, the necessities of life. They, therefore, above board and in an honest and ethical way, have offered "for sale" in one way or another the product of their brain. I repeat that there has been and is just a little manifested tendency on the part of some to criticise or cast insinuating remarks regarding this class of investigators. This is due more to a misunderstanding of the motives which actuated the course, than to anything else.

That I may be more clearly understood, let me say that I have in mind two great men with productive minds, both members of this society—one living and one dead. Their names are known wherever good dentistry is known. Let us review briefly their work.

Several years ago there was a man in Chicago who was a pioneer in inlay work. He began making inlays by one method or another when others seriously and openly questioned his judgment. He was a man, who, like Tennyson, "dipped into the future far as human eyes could see, and saw," so far as the inlay at least was concerned, "the visions of the world, and all of the wonders that were to be." However, he was a thoroughly practical dentist and, inlay enthusiast that he was, he recognized its weak points, and felt that in order for the inlay to be accepted as routine and general practice, we must have better cements. He, therefore, turned his attention to the making of better and more reliable cements. This took time and money, and to accomplish his purpose, he felt it necessary to give up a lucrative practice and offer his product "for sale." To-day he is recognized as one of our best and most reliable cement manufacturers. Who would dare say that dentistry has not been markedly advanced by the researches of our own Doctor W. V.-B. Ames; and is it just to accuse him of being commercial? But perhaps you will say Doctor Ames quit dentistry and entered the commercial field. Is this necessary?

There was a time when one broach practically had the endorsement of the entire profession. Everyone felt that they must use this broach, though an expensive one. A member of our society went to work and made or had made a broach for one-half the price, that was equal in every respect to the former popular broach. This experience led this man into the manufacture of burs, and dental amalgam alloys, and cements, and into the dental supply business—to the end that today we are buying these materials better and cheaper than they no doubt would be, had it not been for our late and highly respected, Doctor J. N. Crouse. Doctor Crouse did not give up his practice, and no one can justly accuse him of being commercial. The work which he did for dentistry, in more ways than one, will stand as a monument to his memory.

This question, like all real questions, has another side. I will simply refer to it briefly. There is a kind of commercial tendency manifested in our profession today which certainly must be discouraged or checked. It is that to which Doctor Otto U. King referred in an editorial in the *Bulletin* of the Indiana State Dental Society some months ago, as was evidenced at the clinics of the Panama-Pacific Dental Congress; it is that to which Doctor C. N. Johnson more recently referred editorially in the April *Dental Review*. It is that tendency—yes, more than a tendency, that deliberate ''exploiting the people'' by calling nitrous oxide and oxygen—''twilight sleep,'' a porcelain jacket crown ''re-enameling,'' the diagnosing in all mouths a condition of pyorrhea, and the offering of a sure and permanent cure by new, original and seclusive methods. I refer to that group of men who have nothing to offer of real value; those who claim to have a remedy, an article or a method of superior value, known only to them and their Creator, which when tested proves to be fictitious. What the profession must learn is that which is needed in every walk of life—the art of discrimination. Some day written rules must be established or a standard given which must govern men in these matters. When these rules or standards are formulated they must be just and equitable; and the sooner they are established and the line sharply drawn, the better it will be for all concerned. My plea is to check or curb the commercial tendencies of the pirate and the charlatan who prey upon the profession and the laity; but I also urge the importance of offering some encouragement to the man who can do more for dentistry in the field of investigation than he can by sticking closely to his practice.—*Dental Review.*

